# Lyme borreliosis incidence in relation to mammalian abundance, climate, and landscape characteristics in a boreal area

**DOI:** 10.1186/s13071-025-07162-7

**Published:** 2025-12-02

**Authors:** Mahdi Aminikhah, Juha Aalto, Jukka T. Forsman, Hilppa Gregow, Heikki Henttonen, Otso Huitu, Mira H. Kajanus, Erkki Korpimäki, Andreas Lindén, Jukka Ollgren, Hannu Pietiäinen, Jussi Sane, Janne Sundell, Leena Ruha, Yingying Wang, Sami M. Kivelä, Eva R. Kallio

**Affiliations:** 1 Department of Ecology and Genetics, University of Oulu, PO Box 3000, 90014 Oulu, Finland; 2 Finnish Meteorological Institute, Weather and Climate Change Impact Research, P.O. Box 503, 00101 Helsinki, Finland; 3 Department of Geosciences and Geography, University of Helsinki, Gustaf Hällströmin Katu 2a, P.O. Box 64, 00014 Helsinki, Finland; 4 Natural Resources Institute Finland (Luke), Paavo Havaksen Tie 3, 90570 Oulu, Finland; 5 Natural Resources Institute Finland (Luke), 00790 Helsinki, Finland; 6 Department of Biological and Environmental Science, University of Jyväskylä, P.O. Box 35, 40014 Jyväskylä, Finland; 7 Section of Ecology, Department of Biology, University of Turku, Turku, Finland; 8 Division of Health Security, Finnish Institute for Health and Welfare, Helsinki, Finland; 9 Lammi Biological Station, University of Helsinki, Pääjärventie 320, 16900 Lammi, Finland; 10 Faculty of Biological and Environmental Sciences, University of Helsinki, 00014 Helsinki, Finland

**Keywords:** Zoonotic disease, Tick-borne disease, Reservoir hosts, *Borrelia burgdorferi* sensu lato, *Ixodes* spp., Species distribution models

## Abstract

**Background:**

The circulation of tick-borne pathogens is influenced by the availability of ticks, the hosts of ticks and pathogens, and the environmental conditions that affect both the ticks and their hosts. Lyme borreliosis (LB), caused by *Borrelia burgdorferi* sensu lato and transmitted by *Ixodes* spp. ticks, is the most common tick-borne disease in the Northern Hemisphere. Understanding the spatio-temporal dynamics of human LB incidence regarding abundance of ticks and hosts and environmental factors is essential for effective disease risk management.

**Methods:**

We analyzed long-term (1997–2018) and spatially extensive (277 municipalities covering 230,000 km^2^) data on human LB incidence in Finland. Using dynamic species distribution models, we assessed the effects of (i) the abundance of pathogen reservoir hosts used by immature ticks (voles and squirrels), (ii) abundance of the key reproductive hosts for adult ticks (moose and deer), (iii) landscape characteristics, and (iv) climatic variables on the risk of LB.

**Results:**

LB presence and incidence varied across the study area and exhibited a clear increasing trend. While host species showed temporal and regional variation in abundance, their relationships with LB risk were inconsistent. In contrast, environmental variables showed more consistent patterns: increased forest fragmentation, longer growing seasons, and higher humidity were generally associated with elevated LB risk.

**Conclusions:**

Our study suggests that the factors explaining LB epidemiology cannot be generalized spatially but depend on local climate, landscape, and host community. Given the available data, environmental conditions seem to play a more predictable role in LB epidemiology than the estimated abundances of hosts at the municipality level, yet we cannot exclude host abundance effects. Hence, the key to enhancing our understanding of the complex mechanisms underlying the epidemiology of LB and other tick-borne infections is to clarify how tick distribution and abundance respond to alterations in the host community, habitat features, and local climate.

**Graphical Abstract:**

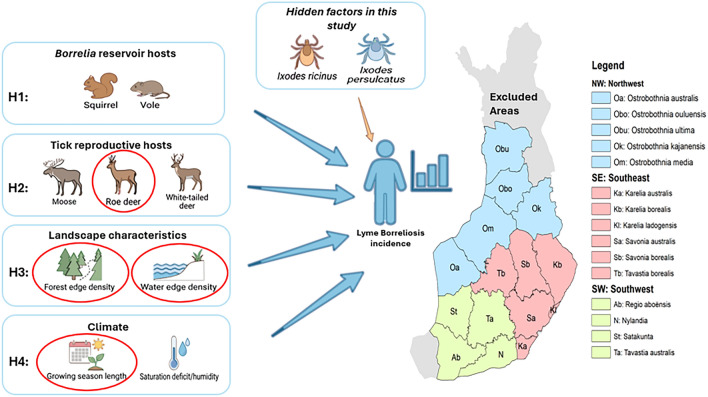

**Supplementary Information:**

The online version contains supplementary material available at 10.1186/s13071-025-07162-7.

## Background

One of the most prevalent tick-borne zoonotic diseases in the Northern Hemisphere is Lyme borreliosis (LB) caused by *Borrelia burgdorferi* sensu lato (s.l.), transmitted by *Ixodes* spp. ticks from wildlife reservoirs to humans [1, 2]. Quantifying the main factors determining LB epidemiology is challenging because of the potentially complex interactions among pathogens, ticks, their hosts, and environmental conditions [3]. Consequently, the spatio-temporal dynamics of LB infections are probably driven by several mutually non-exclusive drivers, but such drivers have rarely been examined over long periods and areas (but see [4]).

*Ixodes ricinus* is the most common tick vector in Europe [1, 5], but *I. persulcatus* is expanding its range and role as a vector in Fennoscandia [6, 7]. Blood meals from hosts are necessary for tick larvae and nymphs to develop to the next developmental stage and adult females to reproduce [8]. Larvae feed predominantly on small mammals and birds, nymphs on medium-sized mammals, while adult females require a blood meal from sufficiently large mammals such as cervids [9]. In Finland, the tick life cycle lasts 2–3 years [10]. Due to differences in their questing behavior [11, 12], *I. ricinus* nymphs and *I. persulcatus* adults are likely responsible for most of the human infections.

Immature life stages of ticks acquire pathogens when feeding on infected hosts and transmit the pathogen further in subsequent blood meals in later life stages [13]. The tick hosts differ in their competence for harboring *Borrelia burgdorferi*. Many small mammals, including mice, voles, and red squirrels, act as competent reservoir hosts for *B. burgdorferi* s.l. to varying degrees [14–18]. Thus, rodent abundance variations translate into variations in both the tick infection prevalence [19–21] and human infection incidence [22–24]. While larger mammals are important hosts for tick reproduction, moose (*Alces alces*), white-tailed deer (*Odocoileus virginianus*), and roe deer (*Capreolus capreolus*) do not harbor *Borrelia burgdorferi* s.l. [17, 25–30]. Hence, the availability of different host types (pathogen reservoir hosts, tick reproductive hosts) is expected to affect the circulation of tick-borne pathogens.

Environmental conditions affect tick abundance and activity [5, 31–33], hence being important for the encounter rate between ticks and humans and consequently the human LB infection risk [34]. In boreal environments, growing season length, temperature, and humidity are important for tick abundance and activity [35]. Locally, the proximity to water bodies may make the environment more suitable for ticks [10]. Landscape characteristics and fragmentation may affect both host and human activity in the environment, which may influence tick abundance and contacts between humans and ticks [36].

In this study, we examine the spatio-temporal dynamics of human LB infections in 277 municipalities in Finland regarding the abundance of different types of hosts and environmental conditions. We apply dynamic species distribution models (DSDMs) to test four hypotheses (H1–H4) to explain LB incidence in humans: (H1) The small mammal hypothesis predicts a positive correlation between the main reservoir host (voles, squirrel) abundances and LB incidence 1 year later. This follows the fact that small mammals infect larval ticks in late summer, and these larvae become nymphs by spring, which can then transmit the pathogen to humans 1 year after the larvae became infected. (H2) The cervids (moose, white-tailed deer, and roe deer) support tick reproduction and, consequently, tick abundance. We expect cervid abundance to increase LB risk with a 2-year time lag, as in Finland, it typically takes 2 years from the time point when an adult female tick feeds and lays eggs until the offspring can infect humans [1]. This is because the eggs need to develop to hatching larvae that then quest for the first (infectious) host and, subsequently, molt to (infectious) nymphs, which quest for a second host. This host can be a human (especially for *I. ricinus*). Alternatively, nymphs feed and molt to (infectious) adult females, which can also infect humans (both *I. ricinus* and *I. persulcatus*). A time lag between human infection and Lyme borreliosis diagnosis is also expected, reflecting the seasonal activity patterns of *I. ricinus* and *I. persulcatus* in Finland [12, 37]. (H3) Landscape characteristics (forest fragmentation and proximity to water bodies) may affect tick abundance through their potential effects on hosts and ticks, hence having positive effects on Lyme borreliosis (LB) incidence. Finally, (H4) a long growing season and humid climate facilitate high tick abundance and activity and are therefore expected to increase LB risk. As the role of the different drivers of LB epidemiology may vary spatially and depend on the dominant tick species (*I. ricinus* and *I. persulcatus*) in the area [7], we examined each of the hypotheses separately in three regions, in addition to analyzing the full study area.

## Methods

### Human LB dataset

**Fig. 1:**
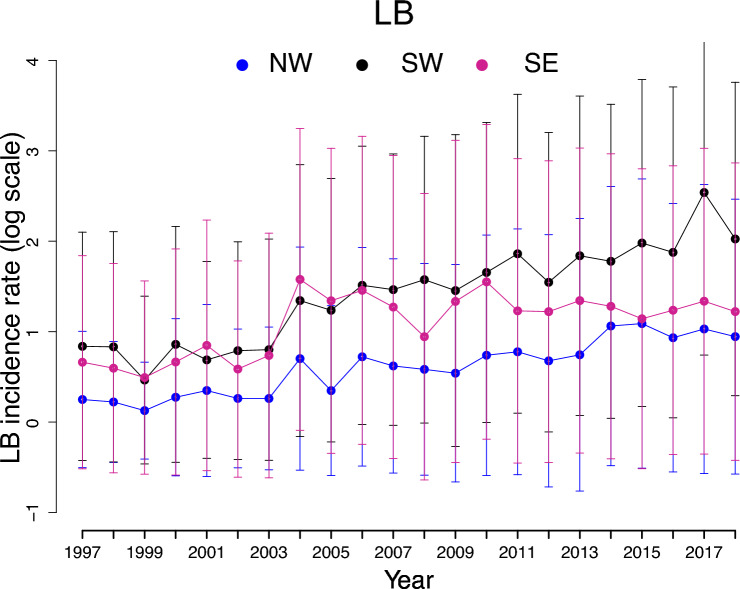
Mean of log LB incidence rates with 95% confidence intervals. Mean values of successive years are connected with dotted lines in each of the three different biogeographical regions (NW: Northwest, SE: Southeast, SW: Southwest). The whiskers indicate the 95% confidence intervals of the region-specific means

We used data on monthly numbers of laboratory-diagnosed LB cases including all laboratory-confirmed cases, excluding cases based on clinical picture only [38] for 1997–2018 (Fig. 1), which are available for each municipality in Finland in the National Infectious Disease Registry (https://sampo.thl.fi/pivot/prod/fi/ttr/cases/fact_ttr_cases). We calculated the LB incidences (infections per 100,000 inhabitants) for “biological years” (see [22]) for each municipality by summing the monthly infections from 1 May in calendar year *t* until 30 April in calendar year *t* + 1 and dividing the resulting total number of cases with the human population size (with multiples of 100,000). At the onset of the biological year in May, the incidence of Lyme borreliosis (LB) cases reaches its annual minimum, and ticks become active following winter dormancy in the study region [10]. Hence, the LB cases diagnosed in winter and early spring (January–April) are allocated to the previous year's tick activity period (April–October) rather than being split into two calendar years.

**Fig. 2:**
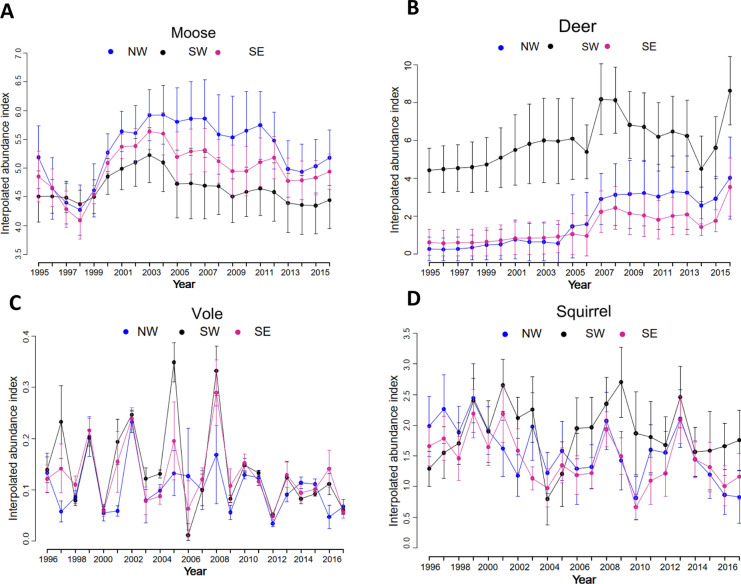
Mean and log abundances of host species with 95% confidence intervals: moose (**A**), deer (**B**), vole (**C**), and squirrel (**D**). Mean values of successive years are connected with dotted lines in each of the three different biogeographical regions (NW: Northwest, SE: Southeast, SW: Southwest). Vole abundance index values are standardized and are not on the log scale. The whiskers indicate the 95% confidence intervals of the region-specific means

**Fig. 3:**
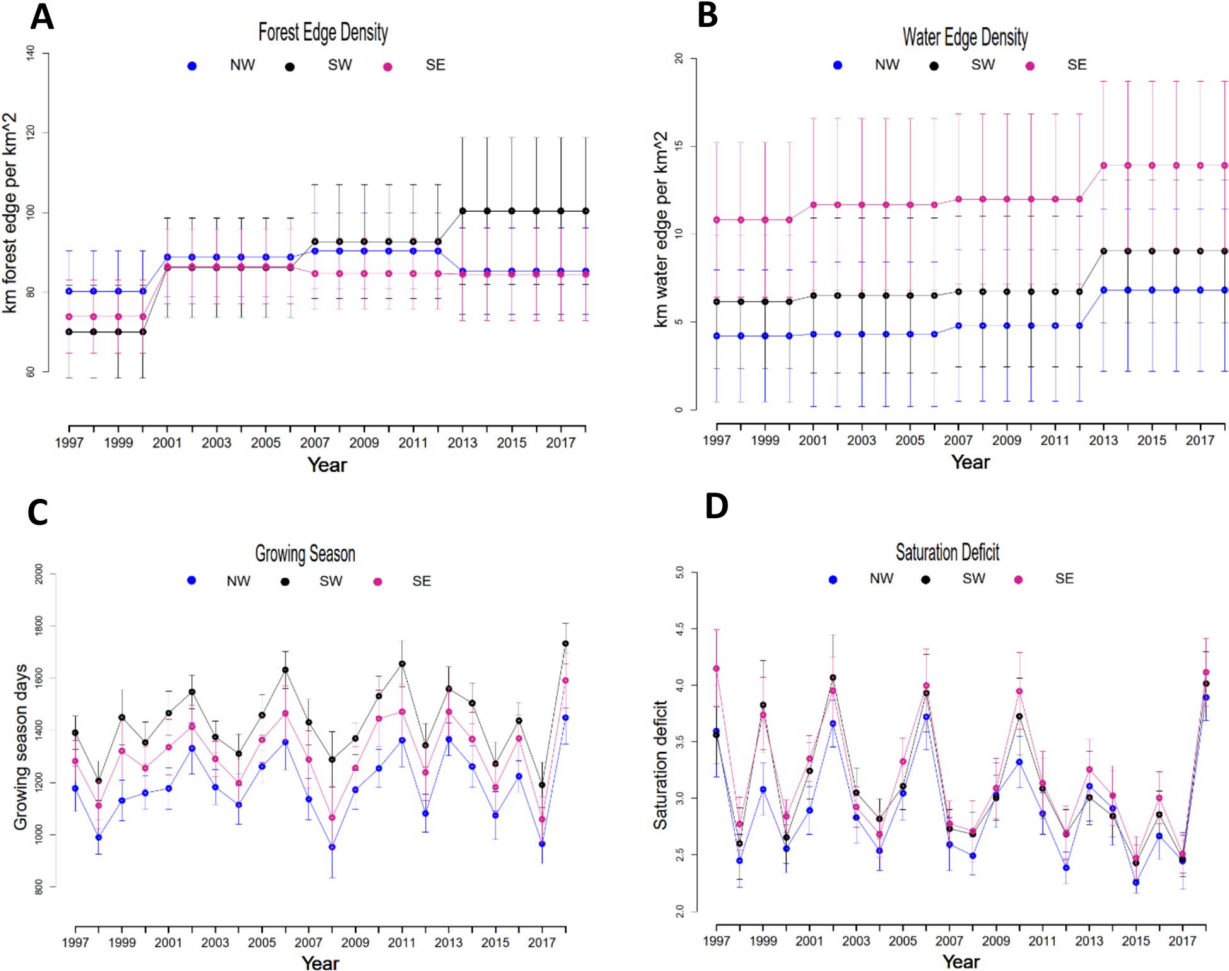
Mean values of climate and land use variables with 95% confidence intervals: forest edge density (**A**), water edge density (**B**), growing season (**C**), and saturation deficit (**D**). Mean values of successive years are connected with dotted lines in each of the three different biogeographical regions (NW: Northwest, SE: Southeast, SW: Southwest). The whiskers indicate the 95% confidence intervals of the region-specific means

We used data from 277 municipalities in Finland, based on their borders in 2017. We excluded the Åland Islands because of a lack of mammalian data (see below) and municipalities in Northern and Northeastern Finland because of the low LB incidence [39], probably because of the scant local tick populations [7]. We divided Finland into three biogeographical regions that differ in the dominant tick species [7], host abundances (Fig. 2), and environmental characteristics (Fig. 3): Northwest (NW), Southwest (SW), and Southeast (SE) (see Supplementary methods I, Fig. S1). We ran the analyses for the three areas separately as well as the entire study area (NW, SW, and SE combined) to determine whether there are common drivers of LB at different scales.

### Mammalian host data

Mammalian host data were based on different sources, and for each study species, we selected the source that was likely to provide the most reliable abundance estimates. For white-tailed deer (*Odocoileus virginianus*), roe deer (*Capreolus capreolus*) and red squirrel (*Sciurus vulgaris*), we used winter snow track data for 1995–2016 from the wildlife triangle monitoring scheme with ca. 800 triangles across the country monitored annually (see details in the supplementary methods I; [40]). We first spatially interpolated the abundances of snow-tracked species, separately for each species and year, using the kriging method [41], to generate snow-track index values over a 2 × 2-km grid across Finland, excluding water bodies. We extracted the mean abundance for each species in each municipality each year using zonal statistics. Ordinary kriging interpolation and zonal statistics were conducted in ArcGIS 10.8. We summed the snow-track indices of roe deer and white-tailed deer (= deer), presuming similar effects of both deer species on LB epidemiology.

Moose (*Alces alces*) abundances were estimated based on the hunting data per season [September–December (January 2017–2018)] in each of the 294 local game management areas in each year (1995–2016). The numbers of hunted individuals were converted to a 1 km × 1-km raster map of hunting density (with each grid cell in a game management area getting the same density). Last, the density of moose hunted in each municipality was obtained by calculating the mean of the density raster over the pixels belonging to the area of each municipality.

### Vole abundance

We used vole abundance data from 35 trapping areas across Finland, compiled by Natural Resources Institute Finland (LUKE). Trappings were conducted in both field and forest habitats in spring and autumn from 1995 to 2018 [42]. We used a vole abundance index based on the pooled abundances of the bank vole (*Clethrionomys glareolus*) and the field vole (*Microtus agrestis*), the most abundant rodents in Finland. We calculated the total number of individuals belonging to these two species in the autumn trapping in both habitats, divided by the number of trap nights, to control the variation in trapping effort between trapping sites and years. Autumn was chosen as the vole index as it coincides with the peak in population abundance following the breeding season, offering representative estimate of vole density during the active tick season, which is critical for host-vector dynamics.

**Fig. 4:**
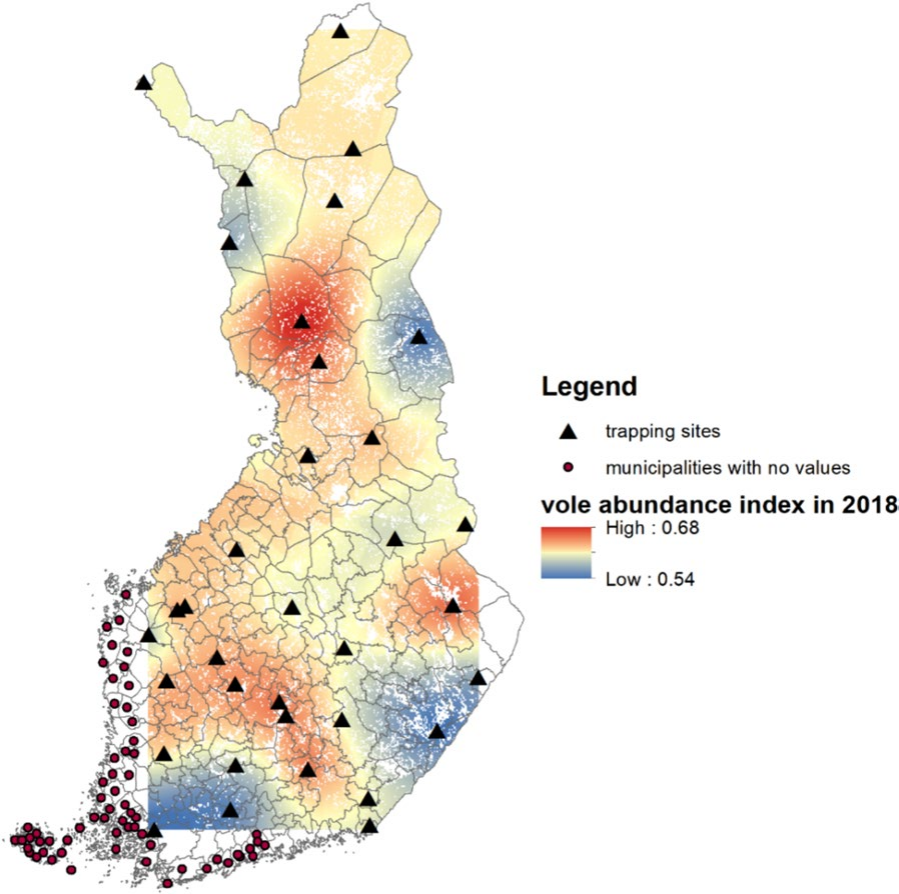
Spatially interpolated vole abundance (colored area) in year 2018 based on 35 trapping areas (triangles). The background map shows municipality borders. Dots show the municipalities that are outside of the grid of vole trapping areas. For these municipalities, vole abundance was estimated to be the same as the nearest observed or interpolated municipality

To allow meaningful comparisons across sites due to variation in habitat quality between trapping sites, we standardized the vole abundance data across years within each site by using z-scores (mean = 0, SD = 1). This approach aligns with the original methodology in [43] and avoids artificial homogenization as well as potential biases due to habitat variation across sites. Vole abundance was then spatially interpolated (see above) to provide estimations for the entire country and then extracted for the municipality level (see Fig. 4). Because the population dynamics of small mammals are synchronous over large areas, this approach facilitated deriving realistic vole abundance estimates for the municipalities, despite the relatively sparse network of vole trapping areas [44].

### Landscape characteristics

For each municipality, we calculated the water edge density [the total length of all water edge segments per unit area (m/ha)], and the forest edge density [the total length of all forest edge segments per unit area (m/ha)] based on Corine land cover data (obtained from the Finnish Environment Institute (Syke; https://www.syke.fi/fi-FI/Avoin_tieto/Paikkatietoaineistot/Ladattavat_paikkatietoaineistot). Water edge density was used as a proxy for proximity to water bodies, which may influence tick and host activity (e.g. higher humidity, host movement corridors). Forest edge density was used as an index of forest fragmentation, which can positively affect host density and tick survival [45]. Land cover data were updated in 2000, 2006, 2012 and 2018 and were assumed to remain unchanged between the updates. While this assumption is reasonable for water bodies, we acknowledge that forest cover may have changed in some areas between the years with data. However, forest edge density also remained relatively stable between 2000 and 2018, as evidenced by the absence of a statistically significant trend in municipality-specific values during this period. A linear regression analysis yielded a slope estimate of 0.0021 (degrees of freedom = 1, *t* = 0.37, *P* = 0.716), indicating that the assumption of temporal stability was reasonable.

### Climate data

We measured growing season as the number of day-degrees above the 5 °C threshold, calculated from mean daily air temperatures [31]. We measured humidity as saturation deficit (*SD;* in units of millimeters of mercury), which was calculated for the main tick activity period from 1 May to 30 September as:

1 $$SD = \left( {1 - \frac{RH}{{100}}} \right) \times 4.9463 \times e^{{\left( {0.0621 \times T} \right)}}$$

where *RH* refers to the mean daily relative humidity in percent and *T* to the daily mean air temperature in degrees Celsius. Low *SD* means high humidity and high *SD* low humidity (see [46]). We used data from 1997 to 2018 for growing season length and saturation deficit based on daily gridded time series at the spatial resolution of 10 km × 10 km [47] and zonal statistics in ArcGIS to extract the annual mean values of each municipality.

### Dynamic species distribution model for LB presence and incidence

We analyzed spatio-temporal variation in LB incidence and its drivers with dynamic species distribution models (DSDM) by using the Vector-Autoregressive Spatio-Temporal model (VAST) with an R package VAST (version 2.0.1; see details in the supplementary methods III), enabling the analyses of spatially and temporally autocorrelated zero-inflated data by using the generalized linear mixed model framework [48, 49]. These analyses were conducted in R version 3.5.2 [46]. Although originally developed for modeling fish biomass, VAST has been successfully applied to community dynamics of terrestrial systems, too [50–52], and here we adapt it to model LB incidence as a spatially structured ecological process. Human LB incidence was the response variable, and the hypothesis-specific variables (host animal abundances, landscape, and climate characteristics) were included as covariates in the models. We used natural logarithmic transformation for abundances of all mammal species, except for voles, as the untransformed vole abundance distribution was approximately symmetric.

We specified the VAST model as a conventional delta model including two linear predictors; the first linear predictor explains the presence of LB (i.e. the probability that LB is present in a municipality; modeling of excess zeroes), and the second linear predictor explains the incidence of LB conditional on presence (i.e. modeling non-zero observations; see supplementary methods II; [48]). We used gamma distribution for incidence conditional on the presence (see Table S1). Spatial and spatio-temporal random effects were modeled by Gaussian random fields, approximated with stochastic partial differential equations as implemented in the R-INLA package [53]. Temporal autocorrelation was modeled as a first-order autoregressive process and spatial autocorrelation with the Matèrn correlation function (see details in the supplementary methods II).

The maximum likelihood estimates of fixed effects were derived using a non-linear optimizer with the Template Model Builder package (TMB; [54]) within R version 3.5.2 [55]. The joint likelihoods of random effects and data were used for predicting random effects in relation to the maximum likelihood estimates of the fixed effects (see [52] for details). We used standard errors that TMB derived by a generalization of the delta method to derive confidence intervals for fixed effects. We considered all parameters whose 99% confidence intervals did not encompass zero to be statistically significant (see below). Furthermore, we used joint Wald tests to assess the effects of each covariate on presence and incidence (i.e. on their respective linear predictors), with the null hypothesis that the two coefficients were simultaneously zero. The Wald statistic (*W*) was derived as *W* = **b**^T^ **C**^–1^ **b**, where the two coefficients for presence and incidence are arranged in vector **b** (**b**^T^ being its transpose), and **C**^–1^ is the matrix inverse of their variance-covariance matrix, which in turn was derived from the full variance-covariance matrix of all parameters (an output of the VAST model). Under the null hypothesis, *W* is χ^2^-distributed with two degrees of freedom.

To assess each hypothesis (H1–H4), we ran a VAST model explaining LB incidence in the human population, with the hypothesis-specific covariates included separately for each region (NW, SW, SE, and the entire area) (see Fig. S1). Separate models were fitted for each hypothesis to avoid overfitting and problems due to collinearities between abundances of different host species. H1 was tested by using vole and squirrel abundances as covariates. H2 was tested by including the abundance of moose and deer as covariates. To test H3, water and forest edge densities were included as covariates in the model, and H4 was tested by including growing season length and saturation deficit as covariates. The statistical significance level was adjusted for multiple independent tests (four hypotheses) by considering a risk level of 0.05 (α) and calculating 0.05/4 = 0.0125 risk level instead of 0.05. Consequently, we considered only results with *P* < 0.0125 as statistically significant.

## Results

We first explored the raw data to understand regional differences in Lyme borreliosis (LB) dynamics. Descriptive summaries of LB incidence, host abundances, and environmental variables across the three regions (NW, SW, SE) from 1995 to 2018 are presented in Figs. 1, 2, 3, 4. Moose populations increased notably in the early 2000s, followed by a gradual decline (Fig. 2A), while deer abundance increased in all regions (Fig. 2B). Vole and squirrel populations fluctuated intensely, with voles demonstrating cyclical dynamics (Fig. 2C, D). Forest edge density showed only modest increases, indicating relative stability of forest fragmentation (Fig. 3A). In contrast, water edge density remained stable in NW and SW but was consistently higher and gradually increasing in SE (Fig. 3B). Climatic variables also changed: growing season length consistently varied across regions (Fig. 3C), while saturation deficit followed a cyclical temporal pattern (Fig. 3D). These climate and landscape changes coincided with a steady rise in Lyme borreliosis (LB) incidence rates, particularly in the SW and SE (Fig. 1), suggesting that regional ecological and climatic shifts may be contributing to the increasing risk of LB.

We examined each hypothesis (H1–H4) concerning LB epidemiology separately, and this was repeated for the three regions and the entire study area.

**Table 1:** Joint Wald statistics (*W*; calculated across the two linear predictors of the VAST models) for hypothesis-specific assessment of factors potentially affecting LB incidence in the human population

Hypothesis	Parameter	Estimate							
		NW		SW		SE		Entire study area	
		*W*	p	*W*	p	*W*	p	*W*	p
H1	Squirrel (1-year lag)	0.0832	0.959	1.668	0.434	0.212	0.899	0.706	0.702
	Voles (1-year lag)	3.731	0.154	0.938	0.625	0.895	0.639	2.153	0.340
H2	Moose (2-year lag)	1.902	0.386	2.514	0.284	0.104	0.949	6.888	0.032
	Deer (2-year lag)	0.271	0.873	1.143	0.564	**10.546**	**0.005**	4.962	0.084
H3	Forest edge density	1.051	0.591	**1.704**	** < 0.001**	6.666	0.036	1.905	0.385
	Water edge density	1.600	0.449	**19.208**	** < 0.001**	1.390	0.499	3.152	0.206
H4	Saturation deficit	1.923	0.382	4.794	0.091	2.0883	0.351	7.637	0.022
	Growing season	3.939	0.139	2.598	0.272	0.876	0.645	**10.700**	**0.005**

The assessment is repeated for Northwest Finland (NW), Southwest Finland (SW), and Southeast Finland (SE), as well as the entire study area (NW + SW + SE; see Fig. 4). Note that the significance assessment was corrected for multiple testing, and only effects with *p* < 0.01 are considered significant (shown in bold). The estimates for the presence and incidence of LB of statistically significant parameters are provided in Table S2

**Fig. 5:**
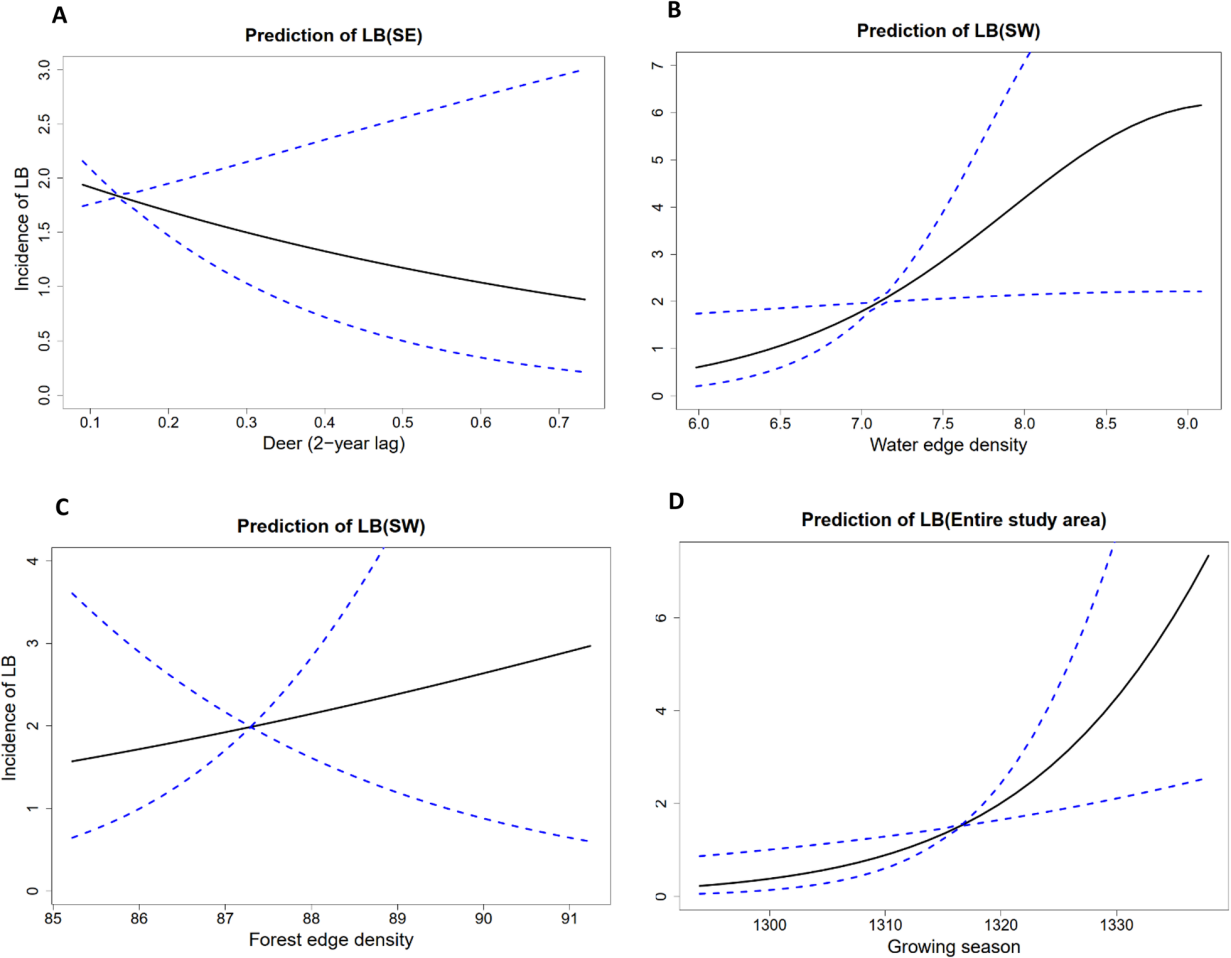
Incidence of LB regarding deer (2-year lag) (**A**), water edge density (**B**), forest edge density (**C**), growing season (**D**). Fitted regression lines (unbroken lines) and their 95% confidence intervals (blue dashed lines) are also presented based on VAST models separately for the different biogeographical regions (SE: Southeast, SW: Southwest). The coefficients used in drawing the regression lines are based on product of the fitted occurrence probability and incidence according to the VAST model. This figure only shows the variables that were significant in the Wald joint test. In each plot, lines are drawn by keeping the other covariates in the model fixed to their mean values

## Hypothesis H1

### ***Borrelia* reservoir hosts.**

Variation in LB incidence was not explained by the abundances of voles or squirrels 1 year earlier in either any of the three regions or the entire study area (Table 1). Hence, these small mammals seem not to be strong predictors of temporal or spatial LB risk at the municipality level.

## Hypothesis H2

### **Tick reproductive hosts.**

Deer (roe and white-tailed deer) abundance 2 years earlier was negatively associated with predicted LB incidence in SE Finland (Table 1 and S2, Fig. 5A). This effect was not found in the other regions or in the entire study area (Table S2, Fig. S3).

## Hypothesis H3

### **Landscape characteristics.**

Water edge density was only positively associated with expected LB incidence in SW Finland (Fig. 5B; Table 1). Forest edge density was positively associated with LB incidence in SW Finland, while there was no association in NW Finland (Fig. 5C; Tables 1 and S2).

## Hypothesis H4

### **Climate.**

The length of the growing season was positively associated with expected LB incidence in the entire study area but not in the sub-regions (Figs. 5D and S2–S3; Tables 1 and S2). Note that the negative association between saturation deficit and expected LB incidence means that expected LB incidence tends to decrease toward dryer conditions (saturation deficit is negatively correlated with relative humidity).

## Discussion

Our results highlight the complexity and spatial variability of ecological and environmental factors influencing Lyme borreliosis (LB) risk in the human population. While we found some support for hypotheses H2 (tick reproductive hosts), H3 (landscape characteristics), and H4 (climatic conditions), the associations were not consistent across regions. Moreover, there was a negative association between deer and LB incidence in one region, being contrary to the predictions of H2. Consequently, the roles of the different hosts as well as landscape characteristics and climate depended on the spatial scale of the investigation and differed among the three studied regions, suggesting that the drivers of LB epidemiology vary spatially (see also [4]).

Contrary to our expectations (H1) [22, 24], rodent abundance did not explain LB incidence variation, suggesting that other reservoir hosts not considered here, such as birds, could be more relevant to LB epidemiology [56]. Although small mammals are generally expected to influence the infection prevalence in ticks, their impact on tick abundance is variable. While some studies suggest limited effects on tick numbers, others have demonstrated a positive association between small mammal populations and tick abundance [19, 57, 58]. In fact, the density of infected ticks, which depends on both tick abundance and their infection prevalence, is important for Lyme borreliosis (LB) epidemiology, and it tends to vary more than infection prevalence in ticks alone [59, 60]. Furthermore, in areas where vole populations show high-amplitude multiannual cycles [61], such as in Fennoscandia, the small mammal species that are the most significant for determining LB prevalence in ticks may change from year to year (e.g. voles in one year and squirrels in the next). In addition, as we have shown earlier [22], the association between LB and vole abundance may not follow expected time lags, potentially partly due to changes in the vole cycles. Also, an insufficient spatial resolution of the vole data in our study could be another reason for the lack of association between vole abundance and LB epidemiology.

Large ungulates (moose, roe deer, and white-tailed deer), serving as tick reproductive hosts (H2), showed some unexpected negative association with LB epidemiology in Southeast Finland. This suggests that a high density of ungulates may reduce LB incidence in humans by reducing infection prevalence in nymphal ticks because of the dilution effect [62, 63]. A recent study, however, shows that the dilution effect of deer on tick infection prevalence is not sufficient to override the amplification effect of deer on tick abundance [64]. Indeed, we expected high deer abundance to increase the LB incidence in Southern Finland, as has been observed with tick-borne encephalitis infection risk in humans [65]. The negative association found in Southeast Finland and the lack of an association in Northwest and Southwest Finland between LB epidemiology and deer abundance suggest that some other drivers are more important for LB epidemiology in these regions [66]. However, deer density was the highest in the SW, where also LB risk was the highest. Thus, further studies are needed to further quantify the role of large ungulates on LB risks especially in regional scales.

Landscape characteristics (H3) were associated with LB incidence in Southwest Finland. Water edge density, which may enhance local humidity and vegetation growth [67], was positively associated with LB incidence. This is consistent with the idea that moist environments favor tick survival and activity. In addition, increasing forest fragmentation was positively associated with LB incidence in Southwest and Southeast Finland, as expected. An earlier study [68] found that forest fragmentation increases LB cases, likely because of higher tick abundance in fragmented deciduous forests. Separately, Moon et al. [69] identified landscape metrics such as forest cover and edge density as strong predictors of LB incidence across varied community contexts. Indeed, forests are important habitats where humans come into contact with ticks.

Climatic conditions (H4) explained LB incidence at the scale of the entire study area. A longer growing season was positively associated with LB incidence, likely reflecting enhanced tick development and activity under warmer conditions [68]. Saturation deficit, a measure of dryness, was negatively associated with LB incidence, indicating that more humid conditions favor tick survival and pathogen transmission.

We used extensive datasets to examine the associations between Lyme borreliosis (LB) incidence in humans and the abundance of different host groups, as well as land use and climate variables. Unexpectedly, we found only limited support for the hypotheses regarding host abundance effects. While certain host or environmental variables were significantly associated with LB incidence, these associations should be interpreted as patterns of covariation rather than evidence of direct causality. It is important to acknowledge the limitations of our data, particularly in terms of spatial and temporal resolution, host detection accuracy, and potential unmeasured confounding factors. Due to these limitations, we cannot exclude the possibility that host abundance effects on LB epidemiology exist at broader spatial or temporal scales, but our analysis may have lacked the sensitivity to detect them. Future studies with finer-scale data and more comprehensive host monitoring may help to clarify these relationships. LB incidence reflects the likelihood of a human encountering and being bitten by an infected tick, a process shaped by a complex interplay of ecological dynamics, environmental conditions, and human behavior. As such, these associations may reflect indirect or mediated effects within a broader system, and caution is considered when drawing conclusions about underlying mechanisms. Bearing in mind that ticks and tick-borne pathogens are especially common in urban areas in Finland [70–73], whereas the host data are collected and thus represent the situation outside cities, there may be a spatial mismatch between the host and human data, which may explain the partially unexpected results.

## Conclusions

Understanding the ecological and environmental factors that influence the maintenance of *Borrelia burgdorferi* s.l. in host communities and its transmission to humans is essential for managing Lyme borreliosis (LB) risk. However, our findings underscore that these drivers are complex, region-specific, and not universally applicable even across Finland, as we did not identify a single factor influencing LB incidence nationwide. Instead, our results point to spatially variable associations shaped by differences in host community composition, landscape structure, and climatic conditions. This highlights the importance of localized approaches to disease risk assessment and management. LB incidence is ultimately determined by human exposure to infected ticks, which is influenced by ecological interactions, human behavior, and environmental context. Future research should aim to elucidate these pathways using finer scale data. To improve our understanding of LB epidemiology and tick-borne diseases more broadly, it is critical to investigate how tick populations respond to changes in host availability, habitat fragmentation, and climate variability. Integrating data on tick abundance, infection prevalence, and human activity patterns will be the key to developing predictive models and effective public health strategies.

## Supplementary Information

Additional file 1.

## Data Availability

The data used in the current analyses are available in an online archive at Zenodo. Aminikhah M et al. [74] Lyme Borreliosis incidence in relation to mammalian abundance, climate, and landscape characteristics in Northern Europe. (10.5281/zenodo.10338820).
