# Correction: Lyme borreliosis incidence in relation to mammalian abundance, climate, and landscape characteristics in a boreal area

**DOI:** 10.1186/s13071-026-07430-0

**Published:** 2026-05-13

**Authors:** Mahdi Aminikhah, Juha Aalto, Jukka T. Forsman, Hilppa Gregow, Heikki Henttonen, Otso Huitu, Mira H. Kajanus, Erkki Lindén, Andreas Lindén, Jukka Ollgren, Hannu Pietiäinen, Jussi Sane, Janne Sundell, Leena Ruha, Yingying Wang, Sami M. Kivelä, Eva R. Kallio

**Affiliations:** 1 Department of Ecology and Genetics, University of Oulu, PO Box 3000, 90014 Oulu, Finland; 2 Finnish Meteorological Institute, Weather and Climate Change Impact Research, P.O. Box 503, 00101 Helsinki, Finland; 3 Department of Geosciences and Geography, University of Helsinki, Gustaf Hällströmin Katu 2a, P.O. Box 64, 00014 Helsinki, Finland; 4 Natural Resources Institute Finland (Luke), Paavo Havaksen Tie 3, 90570 Oulu, Finland; 5 Natural Resources Institute Finland (Luke), 00790 Helsinki, Finland; 6 Department of Biological and Environmental Science, University of Jyväskylä, P.O. Box 35, 40014 Jyväskylä, Finland; 7 Section of Ecology, Department of Biology, University of Turku, Turku, Finland; 8 Division of Health Security, Finnish Institute for Health and Welfare, Helsinki, Finland; 9 Lammi Biological Station, University of Helsinki, Pääjärventie 320, 16900 Lammi, Finland; 10 Faculty of Biological and Environmental Sciences, University of Helsinki, 00014 Helsinki, Finland

**Correction: Parasites & Vectors (2026) 19:15** 10.1186/s13071-025-07162-7

Following publication of the article, it was brought to the journal's attention that owing to a processing error during production of the article, there was a problem with Fig. 5: panel D was missing from the figure. The figure has since been corrected in the published article [1]. The publisher thanks you for reading this erratum and apologizes for any inconvenience caused by this processing error.
