# Supplementary material for: Lyme borreliosis incidence in relation to mammalian abundance, climate, and landscape characteristics in a boreal area
Source: Parasit Vectors. 2025 Dec 2;19:15. doi: 10.1186/s13071-025-07162-7 (PMC12784504; doi:10.1186/s13071-025-07162-7)
Supplement: Supplementary file 1 — Additional file 1. [file 13071_2025_7162_MOESM1_ESM.docx]

**Supplementary Information for**

**Lyme Borreliosis incidence in relation to mammalian abundance, climate, and landscape characteristics in a Boreal Area**

**Mahdi Aminikhah^a^, Juha Aalto^b,c^, Jukka T. Forsman^d^**, **Hilppa Gregow^b^, Heikki Henttonen^e^,** **Otso Huitu^e^, Mira H. Kajanus^a,f^, Erkki Korpimäki^g^, Andreas Lindén^e^, Jukka Ollgren^h^, Hannu Pietiäinen^i^, Jussi Sane^h^, Janne Sundell^j^, Leena Ruha^d^, Yingying Wang^f^, Sami M. Kivelä^a^ and Eva R. Kallio^f^**

* Corresponding authors: Mahdi Aminikhah and Eva R. Kallio

Email: [mahdi.aminikhah@oulu.fi](mailto:mahdi.aminikhah@oulu.fi); eva.r.kallio@jyu.fi

**This file includes:**

Supplementary methods I: Biogeographical regions

Supplementary methods II: Mammalian host data

Supplementary methods III: Details of the VAST model

Tables S1 and S2

Figures S1 to S3

**Supplementary methods I: Biogeographical regions**

To take into account the possibility that the drivers of human LB incidence variation differ in different regions, we divided Finland into three biogeographical regions (Fig. S3). The distribution of *I. ricinus* and *I. persulcatus* differ within Finland. *I. persulcatus* is a more northern species, while *I. ricinus* is the dominating species in most of southern and eastern Finland [7]. Also, these tick species have highly seasonal activity patterns [8,9]; typically larval ticks that feed in summer year *t*, become nymphs in the year *t*+1 and adults in year *t*+2. The distribution of the mammalian hosts, including cervids (moose, roe deer and white-tailed deer) and small (squirrels and voles) differ between the biogeographical regions. Moreover, there are differences in the land use between the biogeographical regions. For instance, Southwest Finland is dominated by agricultural land and Southeast Finland has a high density of lakes.


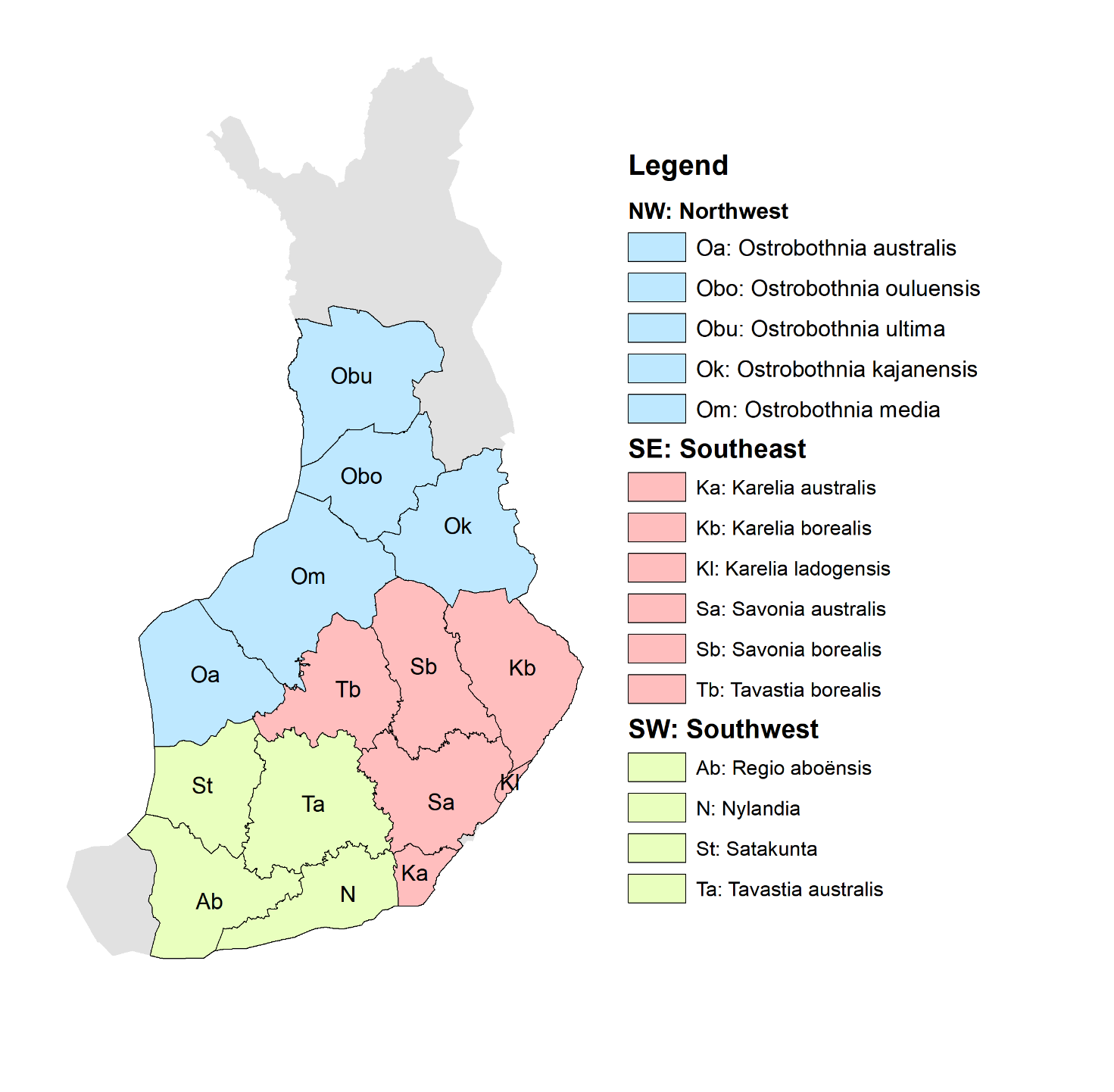
**Figure S1. Map of biogeographical regions used in the study.** The gray color regions were excluded from our study.

| Hypothesis (Model) | Akaike Information Criterion (AIC) | | | | | | | |
| --- | --- | --- | --- | --- | --- | --- | --- | --- |
|  | **NW** | | **SW** | | **SE** | | **Entire Study Area** | |
|  | **gamma** | **lognormal** | **gamma** | **lognormal** | **gamma** | **lognormal** | **gamma** | **lognormal** |
| (1) Borrelia reservoir hosts | 4086.21 | 4092.07 | 11508.24 | 11519.85 | 6353.13 | 6366.33 | 22164.86 | 22170.25 |
|  |  |  |  |  |  |  |  |  |
| (2) Tick reproductive hosts | 4084.37 | 4100.36 | 11509.67 | 11515.32 | 6351.10 | 6355.55 | 22165.96 | 22170.30 |
| (3) Landscape characteristics | 4086.28 | 4090.21 | 11490.49 | 11499.12 | 6346.03 | 6360.22 | 23332.95 | 23332.36 |
| (4) Climate | 4277.21 | 4290.11 | 12101.59 | 12110.28 | 6730.55 | 6740.20 | 23324.01 | 23324.58 |

Table S1. AIC comparison between lognormal and gamma distribution models and selecting the best distribution for fitting VAST model to laboratory-diagnosed LB in a municipality for three regions (Northwest [NW], Southwest [SW], Southeast [SE]) and the entire study area (see Fig. S3 for the specification of the regions).

| **Hypothesis** | **Parameter** | **Estimate** | | | | | |  | |
| --- | --- | --- | --- | --- | --- | --- | --- | --- | --- |
|  |  | **NW** | | **SW** | | **SE** | | **Entire study area** | |
|  |  | P | I | P | I | P | I | P | I |
| **H1** | **Squirrel (1-year lag)** | **-** | **-** | **-** | **-** | **-** | **-** | **-** | **-** |
|  | **Voles (1-year lag)** | **-** | **-** | **-** | **-** | **-** | **-** | **-** | **-** |
| **H2** | **Moose (2-year lag)** | **-** | **-** | **-** | **-** | **-** | **-** | **–0.153**  **(–0.316,**  **–0.010)** | **-** |
|  | **Deer (2-year lag)** | **-** | **-** | **-** | **-** | **-** | **–0.081**  **(–0.145,**  **–0.0167)** | **-** | **-** |
| **H3** | **Forest edge density** | **-** | **-** | **0.170**  **(0.00178,**  **0.577)** | **-** | **-** | **0.136**  **(0.031,**  **0.241)** | **-** | **-** |
|  | **Water edge density** | **-** | **-** | **1.029**  **(0.163,**  **1.895)** | **–0.239**  **(–0.434,**  **–0.0434)** | **-** | **-** | **-** | **-** |
| **H4** | **Saturation deficit** | **-** | **-** | **-** | **-** | **-** | **-** | **-** | **–0.055**  **(–0.098,**  **–0.012)** |
|  | **Growing season** | **-** | **-** | **-** | **-** | **-** | **-** | **0.479**  **(0.054,**  **0.904)** | **0.141**  **(0.026,**  **0.256)** |

Table S2. All the statistically significant parameter estimates and their 99% confidence intervals from hypothesis-specific assessment for the first and second linear predictor of the VAST model explaining the probability of presence (P) and incidence (I) of LB in a municipality for three regions and the entire study area.


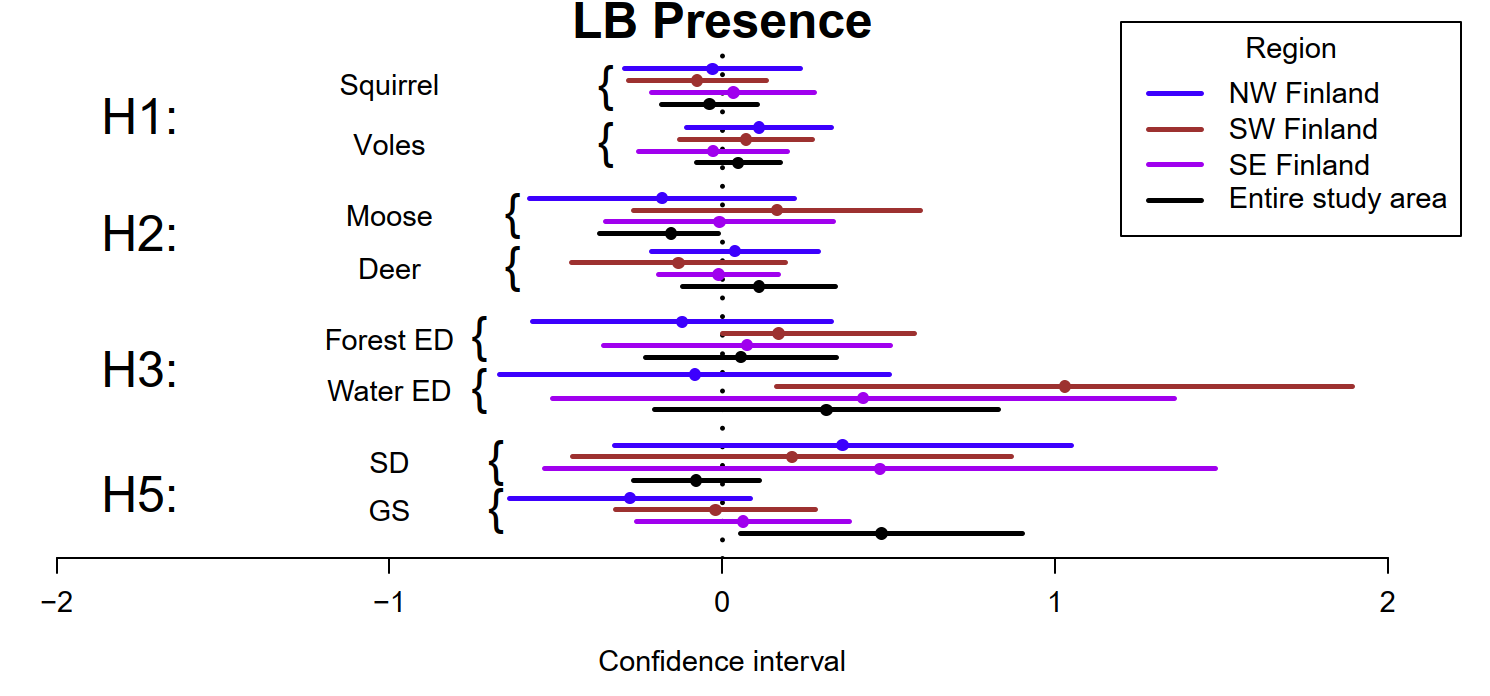


**Figure S2. Fixed effects (circles) and their 99% confidence intervals (whiskers) for the first linear predictor of the dynamic species distribution (VAST) model explaining the logit probability of presence of Lyme Borreliosis** in a municipality for the three regions (NW: Northwest, SW: Southwest, SE: Southeast) and the entire study area (NW + SW + SE) in Finland (different colors in legend). SD and GS refer to the saturation deficit and growing season length, respectively. ED refers to edge density.


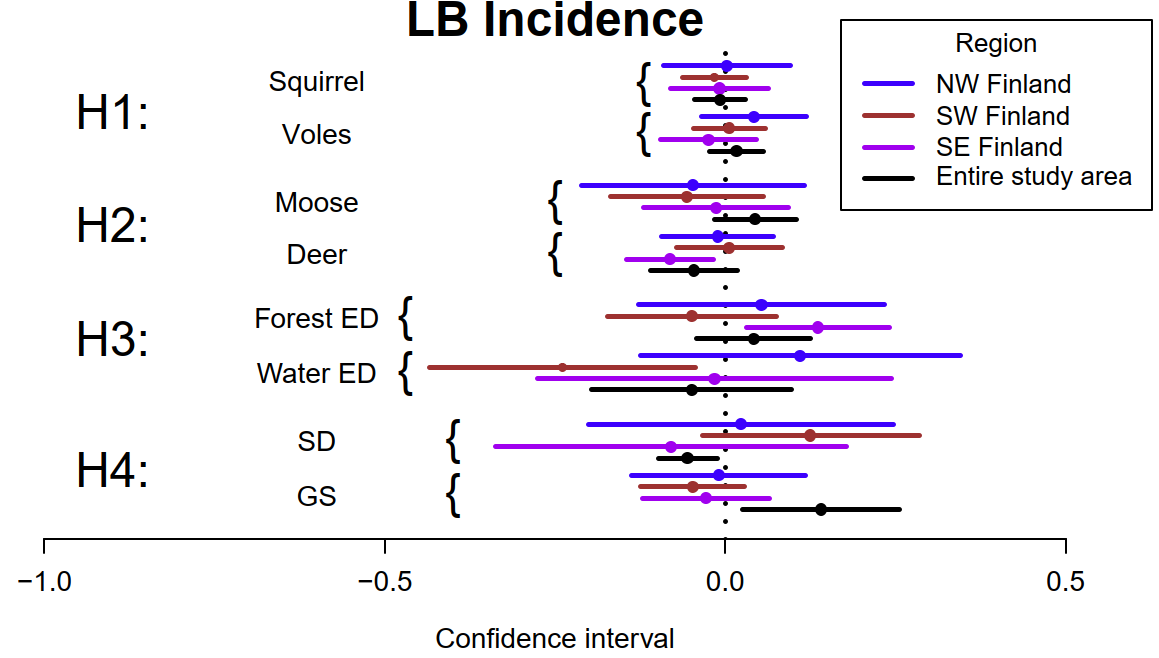


**Figure S3. Fixed effects (diamonds) and their 99% confidence intervals (whiskers) for the second linear predictor of the VAST model explaining the incidence of Lyme Borreliosis** in a municipality for the three regions (NW: Northwest, SW: Southwest, SE: Southeast) and the entire study area (NW + SW + SE) in Finland (different colors in legend). SD and GS refer to the saturation deficit and growing season length, respectively. ED refers to edge density.

**Supplementary methods II: Mammalian host data**

In the annual wildlife triangle winter count, snow track data on 36 (game) animal species are collected by voluntary hunters in winter time (January 15–February 28 or March 15) [1]. The wildlife triangle censuses are done along 12 km long transects located in boreal forest landscape and shaped as equilateral triangles. In addition, we included field triangle censuses, which are implemented using the same methods along 6 km triangle-shaped transects located in a mosaic of agriculture-dominated landscapes, with elements of settlement and forests. The abundances are related to the distance censused and the days during which new snow-tracks may have accumulated (in practice days since last snowfall), resulting in a snow-track index (unit: number of tracks/10 km/day). In total, ca. 800 triangles across the country are monitored annually.

For vole data, each trapping consisted of on average 260 trapping nights per site, the traps being checked once a day [2]. Trapping sites in each location remained largely constant across the years, but sites occasionally had to be changed due to logging or agricultural practices. In these cases, a new site was selected as close as possible to the original site, in a matching habitat.

Due to the interpolation method, spatial interpolation is limited by data coverage, as there were areas outside the grid of vole trapping localities, especially on the coastal areas in Western and Southern Finland. For municipalities in these areas (see Fig. S1), vole abundance was estimated to be the same as in the nearest observed or interpolated municipality. This is justified as vole population fluctuations are spatially synchronous across large geographic areas, spanning up to several hundreds of kilometers [3].

**Supplementary methods III: Details of the VAST model**

The probability distribution of data was specified as

$\Pr\left( b_{st}=B \right)=\left\{ \begin{aligned} 1-p_{st} if B=0 \\ p_{st}\times gamma\left\{ B | r_{st}, \sigma^{2} \right\} if B> 0 \end{aligned} \right.$(1)

where *p_st_* is the probability that LB occurs in municipality *s* in year *t*, and *r_st_* is the incidence of LB given that it occurs in municipality *s* in year *t*. Residual variance of the gamma-distributed LB incidence is *σ*^2^. The logit link function was used for the presence probability of LB, *p_st_*:

$p_{st}=\mathrm{logit}^{-1}(\eta_{1})$ (2)

where *η*_1_ is the first linear predictor of the model (i.e. the linear predictor for presence probability). We used logarithmic link function for LB incidence, given LB presence, from which it follows that

$r_{st}=exp(\eta_{2})$ (3)

where *η*_2_ is the second linear predictor of the model (i.e. the linear predictor for incidence conditional on presence). The linear predictor for presence probability was

$logit\left[ p_{st} \right]=\beta_{1}\left( t \right)+\omega_{1}\left( s \right)+\varepsilon_{1}\left( s,t \right)+ \sum_{i} \gamma_{i}x_{i}(s,t)$ (4)

where $\beta_{1}\left( t \right)$ refers to the intercept for expected presence of LB in year *t*, $\omega_{1}\left( s \right)$ is the spatial variation for expected LB presence among locations *s*, and $\varepsilon_{1}\left( s,t \right)$ represents spatio-temporal variation for expected LB presence across years *t,* and locations *s*. $x_{i}$is the value of covariate *i* in municipality *s* in year *t*. We set the municipality-specific LB sampling area to one because VAST converts abundances to densities by dividing them with sampling area. This was necessary because incidence is already standardized to the ‘sampling area,’ which is the size of the human population in this case. The intercept *β*_1_(*t*) was estimated as a fixed effect and its temporal autocorrelation was modelled as a first-order autoregressive process (AR1). Parameter $\gamma_{i}$ is the effect of the covariate *i* (*i* = {vole abundance, squirrel abundance, moose abundance, deer abundance, water edge density, forest edge density, growing season length, saturation deficit}) on presence of LB. Each covariate was used to explain both the variation in LB presence and incidence across years and spatial locations. The linear predictor for incidence conditional on presence was then:

$log\left[ r_{st} \right]=\beta_{2}\left( t \right)+\omega_{2}(s)+\varepsilon_{2}\left( s,t \right)$+$\sum_{i} \delta_{i}x_{i}(s,t)$ (5)

where $\delta_{i}$ refers to the effect of covariate *i* on LB incidence given predicted presence, and other parameters are defined similarly as in the first linear predictor, subscript 2 referring to the second linear predictor.

Gaussian random fields were used for modelling spatial ($\omega_{i}\left( s \right)$; *i* = 1, 2) and spatio-temporal ($\varepsilon_{i}\left( s,t \right)$) random effects on expected LB incidence (i.e. the product of the two linear predictors) at the locations of 277 spatial knots. Gaussian random fields were approximated with stochastic partial differential equations [4] by using the R-INLA package [5]. Each random field had a variance of 1 and followed multivariate normal distribution with mean of zero and variance of $\sigma_{\omega}^{2}$ and $\sigma_{\varepsilon}^{2}$ for spatial $\omega_{i}\left( s \right)$ and spatio-temporal variation $\varepsilon_{i}\left( s,t \right)$ (*i* =1, 2), respectively

$\omega_{i}\left( s \right)\sim MVN\left( 0,\sigma_{\omega}^{2}\boldsymbol{R} \right)$ (6)

$\varepsilon_{i}\left( s,t \right)\sim\left\{ \begin{aligned} MVN\left( 0,\boldsymbol{R} \right) \text{if }\text{t = }t_{1} \\ MVN(\rho_{\varepsilon}\varepsilon(s,t-1\boldsymbol{)},\sigma_{\varepsilon}^{2}\boldsymbol{R})\text{ }\text{ }\text{ if}\text{ }\text{t > }t_{1} \end{aligned} \right.$ (7)

The spatial covariance matrix, $\boldsymbol{R}$, between location *s* and *s+*1 was scaled with variances of $\sigma_{\omega}^{2}$ and $\sigma_{\varepsilon}^{2}$. Spatio-temporal variation (*ε*) was estimated as a random effect following the Matérn correlation function across space (for more details, see [6]). The smoothness parameter of the Matérn correlation function, *v*, was fixed to one. Parameter $\rho_{\varepsilon}$ is autocorrelation for spatio-temporal covariation.

**REFERENCES**

1. Helle P, Ikonen K, Kantola A. 2016 Wildlife monitoring in Finland: online information for game administration, hunters, and the wider public1. *https://doi.org/10.1139/cjfr-2015-0454* **46**, 1491–1496. (doi:10.1139/CJFR-2015-0454)

2. Korpela K, Helle P, Henttonen H. 2014 Predator–vole interactions in northern Europe: the role of small mustelids revised. *Proceedings of the Royal Society B: Biological Sciences* **281**.

3. Sundell J, Huitu O, Henttonen H, Kaikusalo A, Korpimäki E, Pietiäinen H, Saurola P, Hanski I. 2004 Large-scale spatial dynamics of vole populations in Finland revealed by the breeding success of vole-eating avian predators. *Journal of Animal Ecology* **73**, 167–178. (doi:10.1111/J.1365-2656.2004.00795.X)

4. Lindgren F, Rue H, Lindström J. 2011 An Explicit Link between Gaussian Fields and Gaussian Markov Random Fields: The Stochastic Partial Differential Equation Approach. *J R Stat Soc Series B Stat Methodol* **73**, 423–498. (doi:10.1111/J.1467-9868.2011.00777.X)

5. Lindgren F, Rue H. 2015 Bayesian Spatial Modelling with R-INLA. *Journal of Statistical Software* **63**, 1–25. (doi:10.18637/JSS.V063.I19)

6. Thorson JT, Barnett LAK. 2017 Comparing estimates of abundance trends and distribution shifts using single- and multispecies models of fishes and biogenic habitat. *ICES Journal of Marine Science* **74**, 1311–1321. (doi:10.1093/icesjms/fsw193)

7. Laaksonen M *et al.* 2017 Crowdsourcing-based nationwide tick collection reveals the distribution of Ixodes ricinus and I. persulcatus and associated pathogens in Finland. *Emerg Microbes Infect* **6**, 1–7. (doi:10.1038/emi.2017.17)

8. Cayol C, Koskela E, Mappes T, Siukkola A, Kallio ER. 2017 Temporal dynamics of the tick Ixodes ricinus in northern Europe: epidemiological implications. *Parasit Vectors* **10**, 1–11. (doi:10.1186/s13071-017-2112-x)

9. Pakanen VM, Sormunen JJ, Sippola E, Blomqvist D, Kallio ER. 2020 Questing abundance of adult taiga ticks Ixodes persulcatus and their Borrelia prevalence at the north-western part of their distribution. *Parasit Vectors* **13**, 384. (doi:10.1186/s13071-020-04259-z)
